# Cholera Toxin Regulates a Signaling Pathway Critical for the Expansion of Neural Stem Cell Cultures from the Fetal and Adult Rodent Brains

**DOI:** 10.1371/journal.pone.0010841

**Published:** 2010-05-26

**Authors:** Andreas Androutsellis-Theotokis, Stuart Walbridge, Deric M. Park, Russell R. Lonser, Ronald D. G. McKay

**Affiliations:** 1 Laboratory of Molecular Biology, National Institute of Neurological Disorders and Stroke, National Institutes of Health, Bethesda, Maryland, United States of America; 2 Surgical Neurology Branch, National Institute of Neurological Disorders and Stroke, National Institutes of Health, Bethesda, Maryland, United States of America; Virginia Commonwealth University, United States of America

## Abstract

**Background:**

New mechanisms that regulate neural stem cell (NSC) expansion will contribute to improved assay systems and the emerging regenerative approach that targets endogenous stem cells. Expanding knowledge on the control of stem cell self renewal will also lead to new approaches for targeting the stem cell population of cancers.

**Methodology/Principal Findings:**

Here we show that Cholera toxin regulates two recently characterized NSC markers, the Tie2 receptor and the transcription factor Hes3, and promotes the expansion of NSCs in culture. Cholera toxin increases immunoreactivity for the Tie2 receptor and rapidly induces the nuclear localization of Hes3. This is followed by powerful cultured NSC expansion and induction of proliferation both in the presence and absence of mitogen.

**Conclusions/Significance:**

Our data suggest a new cell biological mechanism that regulates the self renewal and differentiation properties of stem cells, providing a new logic to manipulate NSCs in the context of regenerative disease and cancer.

## Introduction

The adult mammalian brain retains the ability to generate new neurons in small and physically confined areas, the sub-ventricular zone (SVZ) and the dentate gyrus (DG) of the hippocampus [1], [2], [3], [4], [5], [6], [7], [8]. In addition to a role in cell replacement, endogenous NSC and neural precursor populations expand in response to injury [9], [10], [11], [12], [13], [14] and pharmacological manipulations [15], [16], [17], [18], [19], [20], [21], [22], suggesting a role in sensing the state of the tissue and possibly in its repair.

A role for endogenous NSCs in repair is supported by our previous observations that their expansion by various pharmacological activations is followed by rescue of injured neurons in animal models of stroke and Parkinson's disease [22], [23], [24], [25]. These pharmacological treatments include the Notch receptor ligand Dll4, the Tie2 receptor ligand Ang2, insulin, and an inhibitor of JAK kinase. These factors were selected because they converge onto a signal transduction pathway that is critical for the survival of NSCs and involves phosphorylation of STAT3 on the serine residue and induction of the transcription factor Hes3. Using Hes3 and Tie2 as novel markers of NSCs, we have shown that such cells are present throughout the adult central nervous system and their numbers can be powerfully expanded by pharmacological treatments, with subsequent neuroprotection and behavioral recovery. Given the relatively fast onset of protection, the lack of evidence for newly generated neurons, the expression by NSCs of cytokines that protect neurons (e.g. sonic hedgehog), and the close physical association between the NSCs and neurons, it is possible that the expansion of the endogenous NSC population results in increased neuroprotection by trophic support.

Here we expand the arsenal of pharmacological manipulations that regulate NSC numbers by studying the regulation of the pro-survival pathway by cholera toxin. Cholera toxin is comprised of 5 copies of the B subunit that bind to GM1+ gangliosides on lipid rafts, and a single copy of the A subunit that enters the cell via endocytosis, subsequently increasing cAMP levels. In addition, cholera toxin regulates several proteins of the Endoplasmic Reticulum (ER) stress response and the ER associated degradation (ERAD) pathway [26]. This suggests that cholera toxin can mimic stresses known to activate regenerative responses and may increase the expansion of cultured NSCs. We investigated whether the toxin can modulate the signal transduction pathway that is critical for NSC survival [22], [23], [25] and whether it can promote the expansion of cultured NSCs. Our findings show that cholera toxin regulates the expression and localization of Tie2 and Hes3 and induces powerful expansion of cultured NSCs.

## Results

**Figure 1:**
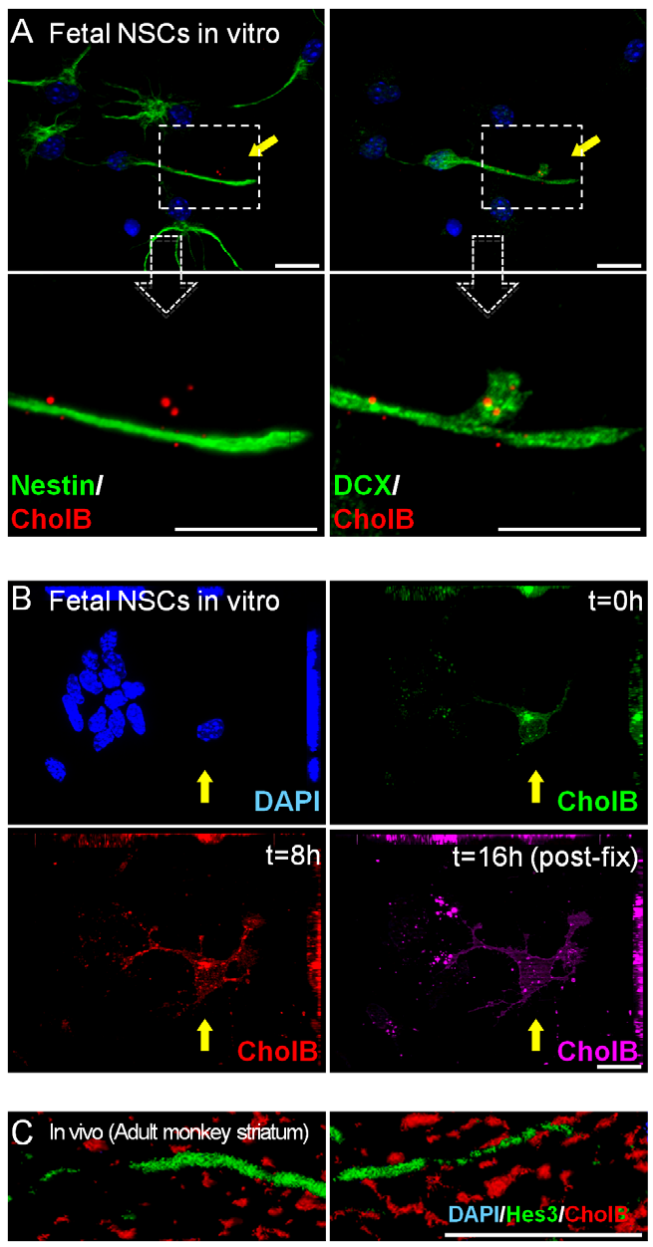
Fetal NSCs exhibit low Cholera toxin B subunit binding. (a) Differentiating DCX+ cells but not fetal NSCs are labeled by CholB in vitro [Magnified images show accumulation of CholB in a DCX+ cell process that is downregulating nestin expression. (b) Multiple pulse-chases by CholB label differentiating cells and not fetal NSCs in vitro. (c) Hes3+ neural precursors in the adult monkey striatum are not labeled by CholB. [Size bars: 20 µm].

Fluorescence-conjugated Cholera toxin B subunit (CholB) recognizes GM1+ gangliosides on lipid rafts and can be used to label cells differentiating towards the neuronal lineage [27], [28], [29]. In established mouse fetal NSC cultures, CholB exhibits no binding or very limited binding to stem cells, but strong labeling of rare contaminating DCX+ neuroblasts and fibroblasts (Figure 1a and Figure S1a,b). GM1+ lipid rafts have been implicated in cytoskeletal re-organization [30]; here we show high concentration of the CholB label on DCX+ cell processes that are selectively down-regulating the stem cell marker nestin (magnification in Figure 1a). Pulse-chase experiments with CholB labeled with different fluorophores showed that throughout a period of 16 h, fetal mouse NSCs show very little labeling, whereas DCX+ cells are continuously receptive to the three fluorophor-CholB used (Alexa488 at t = 0, Alexa594 at t = 8 h, and Cy5 after fixation at t = 16 h) (Figure 1b). In the adult monkey striatum, Hes3+ neural precursors also show very low CholB labeling relative to differentiated cells (Figure 1c and Figure S1c,d). These results show that rodent and primate NSCs and precursors have a small number of lipid rafts relative to differentiated cells.

**Figure 2:**
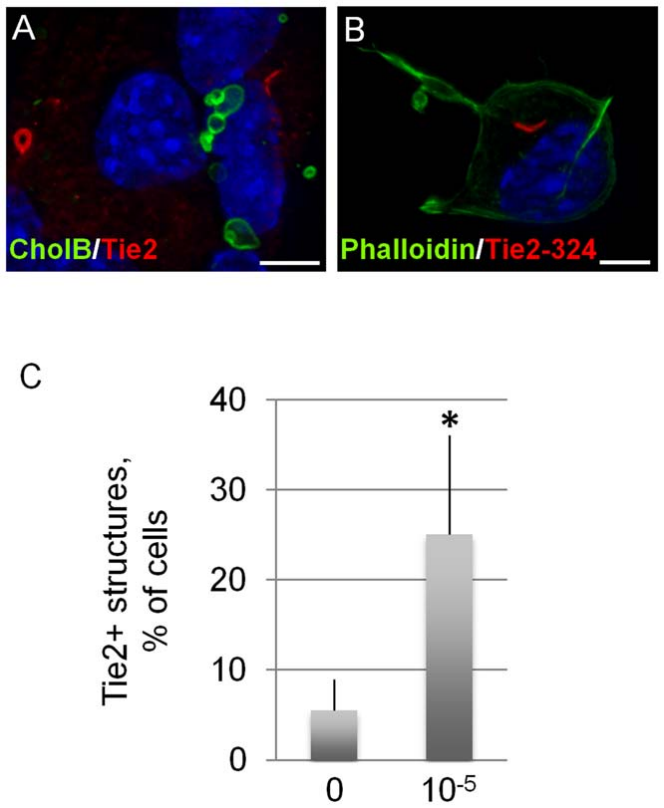
Cholera toxin regulates Tie2 receptors on NSCs. (a, b) High power magnifications of Tie2+ intracellular structures in cultured fetal NSCs. (c) CholAB (2-d treatment) increases the occurrence of Tie2+ structures in adult NSC cultures. [Size bars: 20 µm].

A recent paper cautions that immunocytochemical measurement of ganglioside expression may overestimate their expression (which can be independently determined by biochemical means) [31]. Here we have scored ganglioside expressing cells using a fluorescence conjugated cholera B subunit product (Invitrogen) and high-power confocal microscopy. Cells exhibiting labeling with the characteristic shape of lipid rafts were considered positive, thus avoiding false positives from background signal. We do note, however, that the signal-to-noise ratio was very high as can be seen in Figure 2a and Figure S2. Consistent with this previous study, we find that cultured NSCs exhibit low levels of Cholera toxin B subunit binding, but we also do caution here of the need to perform ganglioside expression studies carefully.

We have recently shown that fetal and adult rodent and primate NSCs in culture as well as a newly identified multipotent neural precursor in the adult brain express the angiopoietin receptor Tie2 and the transcription factor Hes3 [22], [25], [32], [33]. Tie2 and Hes3 immunoreactivity are lost following induction of differentiation. In fetal mouse NSC cultures, an antibody against the C-terminus of Tie2 suggests that the receptor is concentrated in recycling early endosomes (Figure 2a,b), a process regulated by lipid rafts [30]. This observation suggested that treatment with the active form of Cholera toxin (CholAB) may regulate the incidence of Tie2+ endosomes. A 2 day treatment of adult rat SVZ NSCs with CholAB increased the occurrence of Tie2+ structures relative to controls (Figure 2c). Significance p values are given in Table S1.

**Figure 3:**
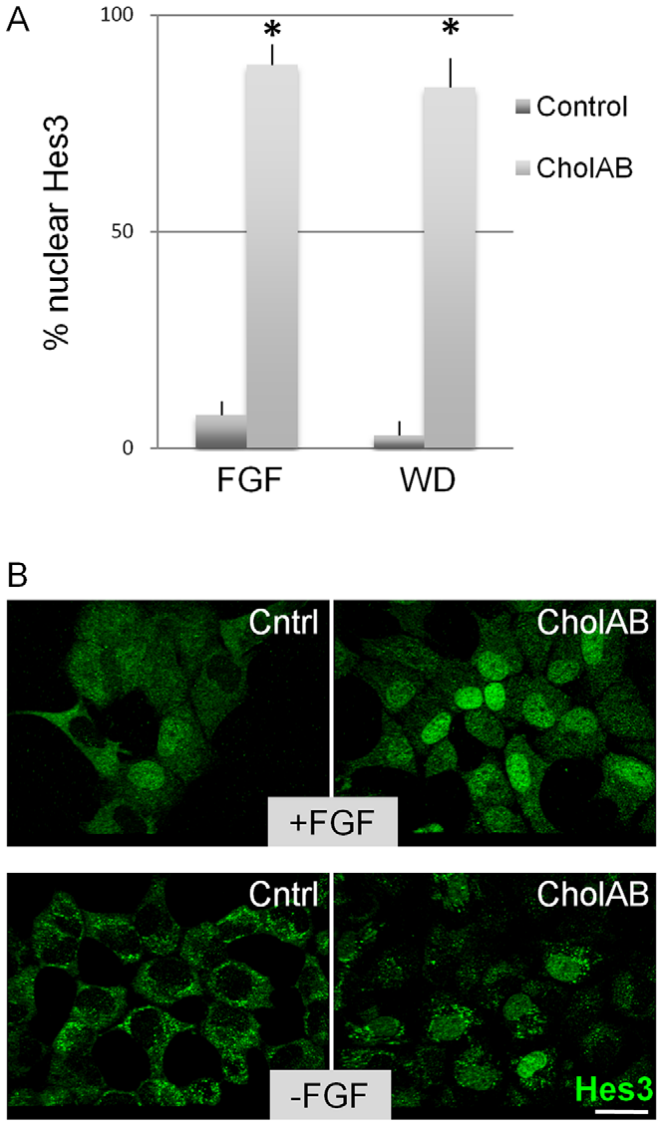
Cholera toxin regulates Hes3 localization in NSCs. (a, b) CholAB (2-d treatment) promotes the nuclear localization of Hes3 in the presence (6 days in FGF2) and absence of FGF2 in fetal NSC cultures. [Size bars: 20 µm].

Downstream of Tie2 signaling is a pathway that induces Hes3 expression and regulates NSC survival [23]. In culture conditions that support the self-renewal of mouse fetal NSCs in vitro (in the presence of FGF2), Hes3 is localized both in the nucleus and the cytoplasm of fetal NSCs (Figure 3a,b; Figure S2; Figure S3), whereas when differentiation is induced (by a 2-day FGF2 withdrawal), Hes3 is only found in the cytoplasm. (Eventually, after approximately a total of 7 days of differentiation, all Hes3 signal is lost [25]). This suggests that nuclear Hes3 identifies the self-renewing, dividing NSC. When fetal NSCs were treated with CholAB for 2 days (following 4 days in FGF2), the nuclear localization of Hes3 markedly increased. This was true both in the presence and the absence of FGF2, suggesting that CholAB may promote the self-renewal state of these cultures and possibly maintain them in the division cycle even after mitogen (FGF2) withdrawal. We note that at earlier times within each passage (i.e. when the cultured cells are proliferating fast) a greater percentage of nuclear Hes3 is observed than at later times as used here (i.e. when the cultures are reaching confluence and the proliferation rates decrease) (data not shown).

**Figure 4:**
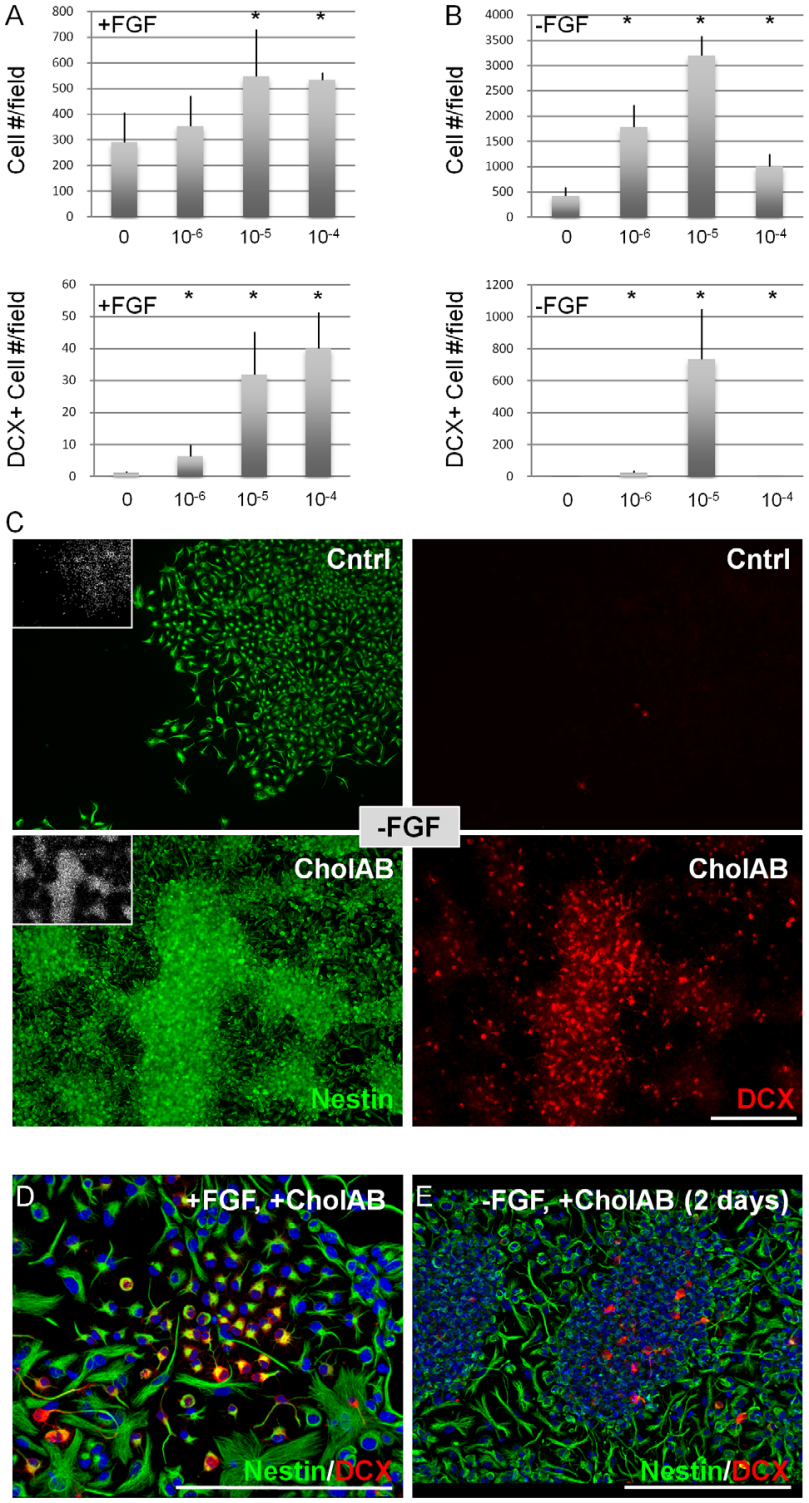
Cholera toxin promotes NSC expansion in vitro. (a) ChAB increases total cell and DCX+ cell number in a dose-dependent manner, in fetal NSC cultures, in the presence of FGF2 (4-d treatment), as well as (b) following FGF2 withdrawal (6-d FGF2 followed by FGF2 withdrawal and addition of ChAB, for 2-days). (c) Confocal projections of fetal NSC cultures described in (b). (d) Fetal NSC cultures expanded in FGF2 (4-days) and then switched to FGF2+/- CholAB – containing medium exhibit expansion of DCX+ early neurons. (e) Fetal NSC cultures expanded in FGF2 (6-days) and then switched to +/- CholAB conditions, in the absence of FGF2 (for 2-days) exhibit expansion of DCX+ early neurons. [Size bars: 100 µm].

**Figure 5:**
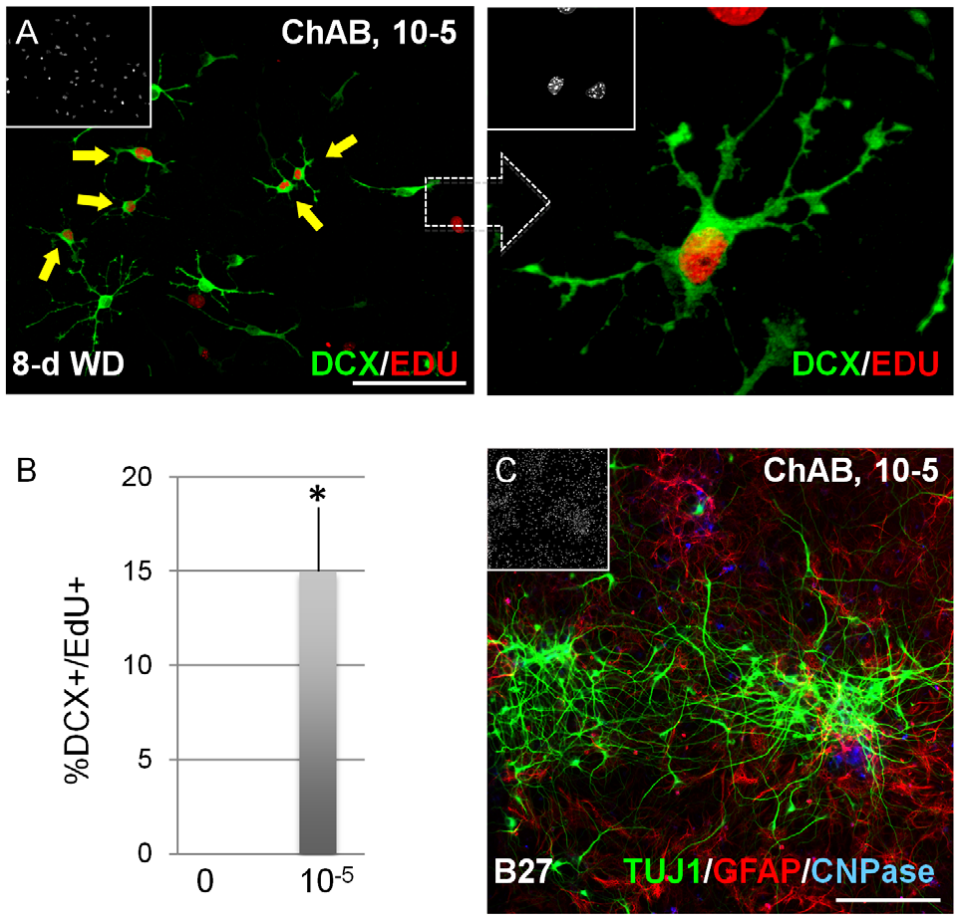
Cholera toxin promotes NSC proliferation in vitro. (a, b) ChAB during an 8-day FGF2 withdrawal period maintains cell division (assessed by incorporation of EdU added on days 7–8); low- and high power confocal projections are shown. (c) ChAB-treated fetal NSC cultures generate neurons, astrocytes, and oligodendrocytes (ChAB was added during FGF2 treatment, 4-days and for another 2-days following FGF2 withdrawal; cells were then switched to B27 medium with added neurotrophins for 7-days). [Size bars: 100 µm].

When mouse fetal NSC cultures were treated with CholAB in the presence of FGF2 (for 4 days), a small but significant increase in total cell number and a large increase in DCX+ cell number was measured (Figure 4a,c). A much greater effect (relative to controls) was obtained when CholAB was added immediately after FGF2 withdrawal for 2 days, as control cells under those conditions exit the cell cycle [34], [35] whereas CholAB – treated cultures increased in cell density (Figure 4b,c). Adherent fetal NSC cultures are highly homogenous, but they also contain small percentages (>1%) of DCX+ neuronal precursors. In the presence of FGF2, CholAB did not induce the acquisition of differentiation markers, but it expanded the DCX+ populations (Figure 4d,e; Figure S4a,b). This is concluded by the increase in DCX+ colony size but not number (data not shown). These results suggest that CholAB – treated cells in the absence of mitogen are still dividing. To address this possibility, fetal NSC cultures were plated in FGF2 for initial expansion, after which FGF2 was removed for 8 days, and during the last two days the thymidine analog EdU was added to the medium to detect any proliferating cells. Under those conditions, no cells in the control groups incorporated EdU, whereas 25%±5 of total cells and 15%±3 of DCX+ cells in the CholAB – treated groups still divided (Figure 5a,b). These data show that CholAB promotes the proliferation of cells in a minimal culture medium (without serum) in the absence of a mitogen. When the CholAB – treated cultures were forced to differentiate (by removal of FGF2, CholAB, and the addition of B27 cell culture supplement and neurotrophins for 7 days), neurons, astrocytes, and oligodendrocytes were generated (Figure 5c), showing that the potential of the culture en masse to generate all three major lineages of neural tissue was not lost.

**Figure 6:**
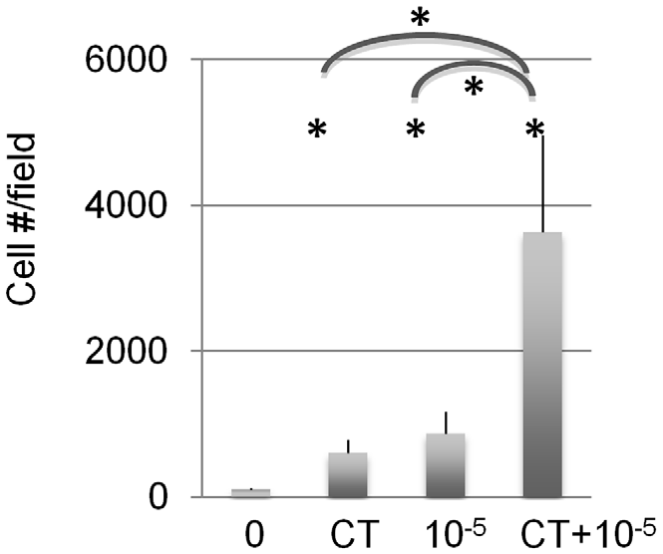
Cholera toxin cooperates with pro-survival factors to expand NSC numbers in vitro. CT and ChAB co-operate to increase fetal NSC numbers (5-day treatment).

We have previously reported a pharmacological treatment that promotes the survival and expansion of fetal and adult NSCs in culture and of adult neural precursors in vivo [25]. This combined treatment (CT) contains the Tie2 ligand Ang2, the Notch ligand Dll4, a JAK kinase inhibitor, and insulin. In these experiments, insulin is contained in the culture medium throughout their duration. We investigated whether the pro-expansion capabilities of CT and CholAB can be combined to establish a new treatment that will maximize NSC number increase. We found that CT and CholAB co-operate to increase mouse fetal NSC numbers in cultures grown for 5 days in the presence of FGF2 (Figure 6). These results show that a combination of pharmacological treatments that affect signal transduction and cell biological processes can greatly enhance the efficiency of primary cell culture.

**Figure 7:**
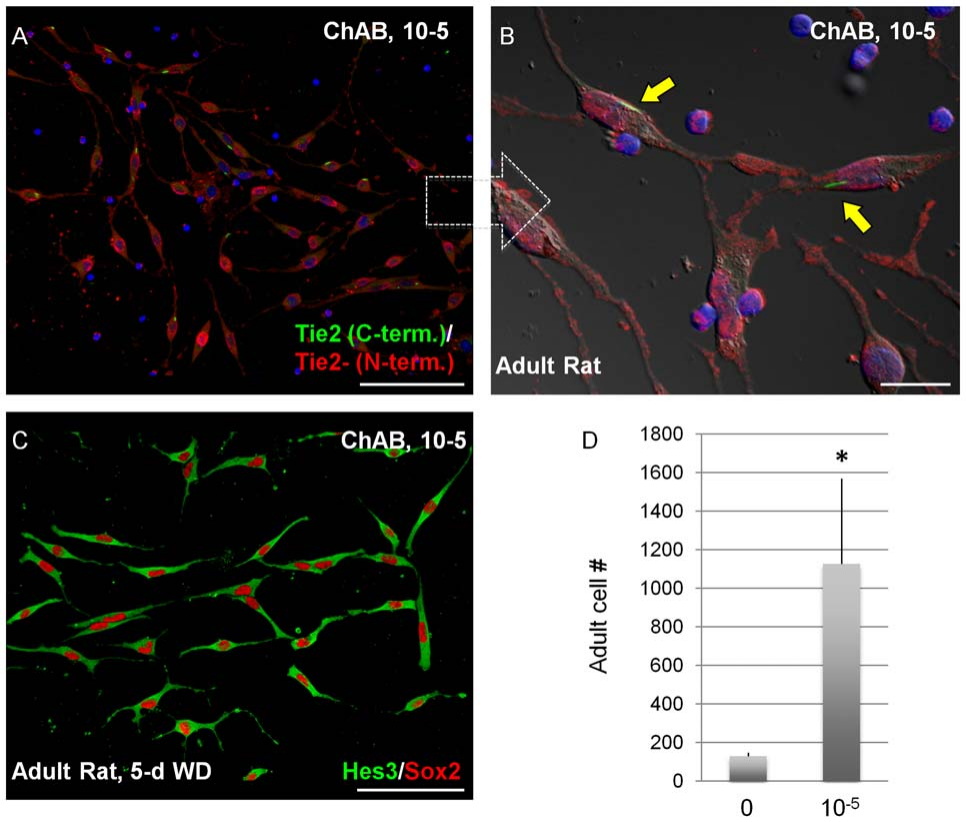
Cholera toxin promotes the expansion of adult NSCs in culture. (a–c) ChAB-treated adult NSCs in vitro co-express Hes3 and Sox2 following 5d FGF2 withdrawal. (d) ChAB promotes the expansion of adult NSC cultures in vitro (7-day treatment). [Size bars: 20 µm].

Cultures of NSCs from the adult rat SVZ express similar markers to their fetal counterparts including Hes3, Tie2 and Sox2 [23], [25]. Here, we show that the expression of these markers is retained in adult cultures 5 days after FGF2 withdrawal, when CholAB is added at the time of FGF2 withdrawal (Figure 7a–c). CholAB treatment also increased Tie2 immuno-reactivity and occurrence of the early endosomal structures that are strongly labeled for C-terminus Tie2 (Figure 7a–c; Figure 2e) and the number of adult NSCs in culture conditions that included FGF2 for 5 days (Figure 7d).

## Discussion

Our results suggest that interference at the level of GM1+ lipid raft function affects many properties of fetal and adult NSCs including the regulation of receptors and transcription factors that are shown to be critical for their survival and expansion, and may provide new targets to manipulate NSCs in normal and cancerous tissue.

Consistent with previous work from other labs [27], [28], [31], our work suggests that NSCs exhibit little Cholera toxin B subunit binding. In fact, at their most self-renewing, multipotent state, soon after plating [35], they exhibit the lowest degree of binding, with many NSCs showing no detectable binding at all. An interpretation of these observations is that the effect of cholera toxin is increasingly more potent to cells as they progress through the process of differentiation. The end effect is a partial return to their more immature state. This is supported by our mechanistic data showing that treatment with the full (active) cholera toxin protein increases immunostaining for the Tie2 receptor and induces the nuclear localization of the transcription factor Hes3, two processes characteristic of self renewing NSCs [22], [23], [24], [25].

In the skin, a similar relation between lipid rafts recognized by cholera toxin and cell maturity has been reported. Stem-like cells in the skin show little cholera toxin binding, whereas more differentiated, transit amplifying cells are enriched in lipid rafts [36], strongly correlating to the neural cells reported here. Intriguingly, human embryonic stem (hES) cells and human induced pluripotent stem (hiPS) cells appear insensitive to cholera toxin treatment [37], suggesting that the reprogramming of skin fibroblasts to the pluripotent state involves the loss of signaling through GM1 ganglioside – containing lipid rafts [and downstream G_s_-coupled G protein –coupled receptor (GPCR) signaling]. hES cells and hiPS cells, instead, are sensitive to pertussis toxin, acting through G_i_ GPCR signaling [37]. These data suggest that different stem cell states operate distinct cell surface receptors leading to specific GPCR signaling pathways and that transitions between stem cell states (forwards and backwards) switch between these pathways.

We have previously elucidated a signal transduction mechanism that is critical to the survival and expansion of neural stem cells in vitro and in vivo. This pathway involves cell surface receptors (Notch, insulin, Tie2) and subsequent up-regulation of the transcription factor Hes3 [22], [23], [24], [25]. Here we show that a cell biology mechanism that is activated by cholera toxin converges into this pathway (at the levels of receptor trafficking and Hes3 localization) to regulate stem cell and precursor cell numbers. Our result showing that cholera toxin maintains cells in a proliferative state in the absence of mitogen demonstrates the importance of this signal transduction/cell biology interaction. It also provides an experimental system to help elucidate the mechanism controlling self renewal in neural stem cells. The additive and powerful effects of receptor activation and cholera toxin treatment suggest an untapped potential to interfere with stem cell numbers in vivo.

Further work based on these discoveries is likely to generate novel treatments that will allow the manipulation of endogenous stem cells in the context of disease. We have previously shown that treatments that increase NSC numbers in vivo also confer neuroprotection and behavioral benefits in models of neurological disease [22], [23], [25]. Recently, we have also shown that the presence of Hes3 marks not only the normal endogenous NSC but also the stem cell compartment in malignant brain tumors [24]. A key finding is that the localization of Hes3 correlates strongly with the proliferative state of stem cells: Quiescent endogenous stem cells throughout the adult brain exclude Hes3 from the nucleus but express it in the cytoplasm; when placed in culture under mitogenic support that promotes proliferation, Hes3 is found in both the cytoplasm and nucleus. Similarly, in human biopsies from Grade IV Glioblastoma multiforme, Hes3 is found in both the cytoplasm and nucleus. Uncovering novel treatments that will affect Hes3 expression and localization will be a new set of tools in regenerative and cancer medicine.

## Materials and Methods

### Ethics statement

#### Vertebrate Experiments (rodents)

All research involving animals was conducted in accordance with NINDS ACUC (National Institute of Neurological Disorders and Stroke/Animal Care and Use Committee) guidelines and after their approval [Animal Protocol ASP#1204-05]. The animals were socially housed and provided with environmental enrichment and novel food items as provided for by the IACUC-approved NHP enrichment program. Potential postoperative pain was treated with ketoprofen IM, given preemptively at the time of surgery, and for 2 days post-operatively. Prophylactic antibiotic treatment and nursing care were also provided.

#### Non-Human Primate Experiments

The study was conducted in accordance with the National Institutes of Health Guidelines on the use of animals in research and was approved by the Animal Care and Use Committee of the National Institutes of Neurological Disorder and Stroke [Animal Protocol ASP#1294-08; “Convection-enhanced delivery of stem cell inducing compounds into the primate brain”]. The animals were socially housed and provided with environmental enrichment and novel food items as provided for by the IACUC-approved NHP enrichment program. Potential postoperative pain was treated with ketoprofen IM, given preemptively at the time of surgery, and for 2 days post-operatively. Prophylactic antibiotic treatment and nursing care were also provided.

### Cell Culture

E13.5 neural stem cells were grown as previously described [34], [38]. Cells were expanded in serum-free DMEM/F12 medium with N2 supplement and FGF2 (20 ng/ml) for 5 days under 5% oxygen conditions and were re-plated fresh or from frozen stocks at 1,000–10,000 cells per cm^2^. FGF2 was included throughout our experiments, unless otherwise stated. Adult rat (3–6 months old) or adult mouse (2–4 months old) SVZ Neural Stem Cell (NSC) cultures were grown in the same medium as the fetal cultures.

### In vivo Experiments – monkeys

#### Preparation of Gadolinium-DTPA

Clinical grade gadolinium-DTPA (Berlex; Pointe-Claire, Quebec) was used in all infusions. The gadolinium-DTPA was diluted in PBS solution to a 1 mM concentration.

#### Convective Co-Infusion of Growth Factors and Gadolinium-DTPA

Six (two controls, two treated with Dll4, and two treated with Ang2) adult primates (*Macaca mulatta*) underwent convection enhanced delivery (CED) co-infusions to the right striatum. Each animal received 120 microliters of aCSF, Dll4 (0.4 mg/ml) or Ang2 (0.2 mg/ml) in PBS with BSA carrier (0.1%) and gadolinium-DTPA (1 mM). The animals were sedated, intubated and placed under isoflurane general endotracheal anesthesia. The animals' temperature, heart rate, oxygen saturation, electrocardiographic responses and end-tidal partial pressure of carbon dioxide were continually monitored. The head of each animal was then secured in a stereotactic frame (model 9-YSTI, Crist Instrument Co., Hagerstown, MD). Using sterile technique, a midline skin incision was made from the anterior to the posterior aspect of the vertex of the skull and self-retaining retractors were used to expose the underlying bone. A 8 mm burr hole was placed in the calvarium over the stereotactically determined entry point above the target area and the underlying dura mater was incised. A 22 gauge fused silica outer guide cannula (outer diameter 0.027 in, inner diameter 0.02 in, from Plastics One) was stereotactically placed through the dural opening along the target trajectory to a level 1.5 cm above the desired striatal target. The guide cannula (28 gauge, made of fused silica) was secured to the calvarium with methylmethacrylate. The inner cannula (outer diameter 0.014 in, inner diameter 0.006 in, from Plastics One), after being connected to the infusion apparatus, was placed through the outer guide cannula to the target.

To distribute infusate to the striatum using CED, we used a noncompliant delivery system that is gastight with no dead volume that has been described previously. A Harvard syringe pump (PHD 2000, Harvard Apparatus, Inc., South Natick, MA) was used to generate continuous pressure throughout the infusion procedure. During infusion, pressure was transmitted from the pump to a glass, gastight, infusate-filled Hamilton syringe (250 microliter total volume) that was connected to PE50 thick walled polyethylene tubing (outer diameter 0.050 in, inner diameter 0.023 in) (Plastics One, Roanoke, VA). The tubing was connected to the distal end of the inner infusion cannula and the tip was placed directly into striatum. Infusions were carried out at 0.5 microliters/minute. After the infusion was complete, the cannulas were removed and the animals were euthanized. During the euthanizing process the brain was flushed with heparinized saline and 4% paraformaldehyde solutions prior to being removed. A 1.5 cm thick bilateral coronal slab was removed from the fixed brain, which included both the ipsilateral and contralateral infused hemispheres and was immediately snap frozen for later immunohistochemistry assays.

#### Imaging of Factors and Gadolinium-DTPA Distribution

After placement of the infusion cannula, coronal T1-weighted MR images were obtained to determine the precise location of the infusion cannulas. Once cannula placement was confirmed, infusions were started and T1-weighted MR-images were obtained in 3 planes (sagittal, axial, and coronal; slice thickness 1 mm, 0 mm spacing) using a 3 Tesla MRI-scanner. Images were obtained at approximately 10 to 20 minute intervals until the infusions were complete.

Cell numbers and vascular effects were assessed by immunohistochemistry using several (typically 6) coronal sections taken between +21 mm and +31 mm relative to Ear Bar Zero (EBZ).

### Reagents

We used the following reagents and antibodies: FGF2 (233-FB), mouse Dll4 (1389-D4), fibronectin (1030-FN), human angiopoietin-2 (623-AN), from R&D; JAK Inhibitor I (420099), from Calbiochem; Polyornithine (P-3655), insulin (I9278), Cholera toxin (C8052) from Sigma; BrdU (84447723) from Boehringer; Fluorogold from Fluorochrome, LLC; Alexa-Fluor-conjugated secondary antibodies, AlexaFluor conjugated Cholera Toxin Subunit B (C22841, C22842, C22843), and Alexa-conjugated Phalloidin (A12379) from Invitrogen; DAPI (D-8417) from Sigma, and general chemicals from Sigma. For immunohistochemical staining, we used antibodies against the following markers: nestin (Chemicon, MAB353), Tuj1 (Covance, MMS-435P), GFAP (z0334) from Dako; CNPase (Chemicon, MAB326), BrdU (Accurate, H5903), Sox2 (R&D, MAB2018); Hes3 (25393), Tie2 (sc-324), Tie2 (sc-31266), STAT3 (482), from Santa Cruz; pTie2 (AF2720) from R&D Systems; α-tubulin, Tie2 (R&D, AF313), pTie2 (R&D, AF2720) from (Sigma, T-6074).

### 3D image reconstruction

We used the Volocity 3D imaging software by Improvision (http://www.improvision.com/products/volocity/) and Zeiss Axiovision (http://www.zeiss.com) software on confocal z-stacks.

### Statistical analysis

Results shown are the mean ± s.e.m. Asterisks identify experimental groups that were significantly different (p-value<0.05) from control groups by the Student's t-test (Microsoft Excel), where applicable. The p values shown are within the value allowed after Bonferroni correction. In figure 6e (Rotometry dose curves), the asterisks represent statistical significance as determined by two-way ANOVA analysis (by the GraphPad Prism software). A supplementary table is presented with the p values for each experiment (Table S1).

## Supporting Information

Figure S1 Cholera toxin labels differentiated cells in culture. (a,b) CholB labels differentiated DCX+ cells and fibroblast contaminants in culture; NSCs are identified by Tie2 immunoreactivity. (c) Adult monkey Hes3+ striatal neural precursors are not labeld by CholB. (d) A 30 min pulse of CholB in fetal NSC cultures 2 days after FGF2 withdrawal labels neurons, astrocytes and oligodendrocytes (chased for 6-days). [Size bars: 20 µm].
(3.43 MB TIF)

Figure S2 Cholera toxin promotes the nuclear localization of Hes3. CholAB (2-d treatment) promotes the nuclear localization of Hes3 in the presence and absence of FGF2 in fetal NSC cultures; split channels from confocal projections are shown [Size bars: 100 µm].
(3.79 MB TIF)

Figure S3 Cholera toxin promotes the nuclear localization of Hes3. High-power magnification of the images described in Suppl. Fig. S2 [Size bars: 20 µm].
(2.89 MB TIF)

Figure S4 Cholera toxin inhibits the differentiation of fetal NSCs in culture. (a) Fetal NSC cultures expanded in FGF2 (4-days) and then switched to FGF2+/− CholAB - containing medium do not acquire immuno-reactivity for GFAP or TUJ1. (b) Fetal NSC cultures expanded in FGF2 (6-days) and then switched to +/− CholAB conditions, in the absence of FGF2 (for 2-days) do not acquire immuno-reactivity for GFAP or TUJ1. [Size bars: 200 µm].
(1.34 MB TIF)

Table S1 Significance (p) values.
(0.04 MB DOC)
