# Supplementary figures and images for: Cholera Toxin Regulates a Signaling Pathway Critical for the Expansion of Neural Stem Cell Cultures from the Fetal and Adult Rodent Brains
Source: PLoS One. 2010 May 26;5(5):e10841. doi: 10.1371/journal.pone.0010841 (PMC2877108; doi:10.1371/journal.pone.0010841)

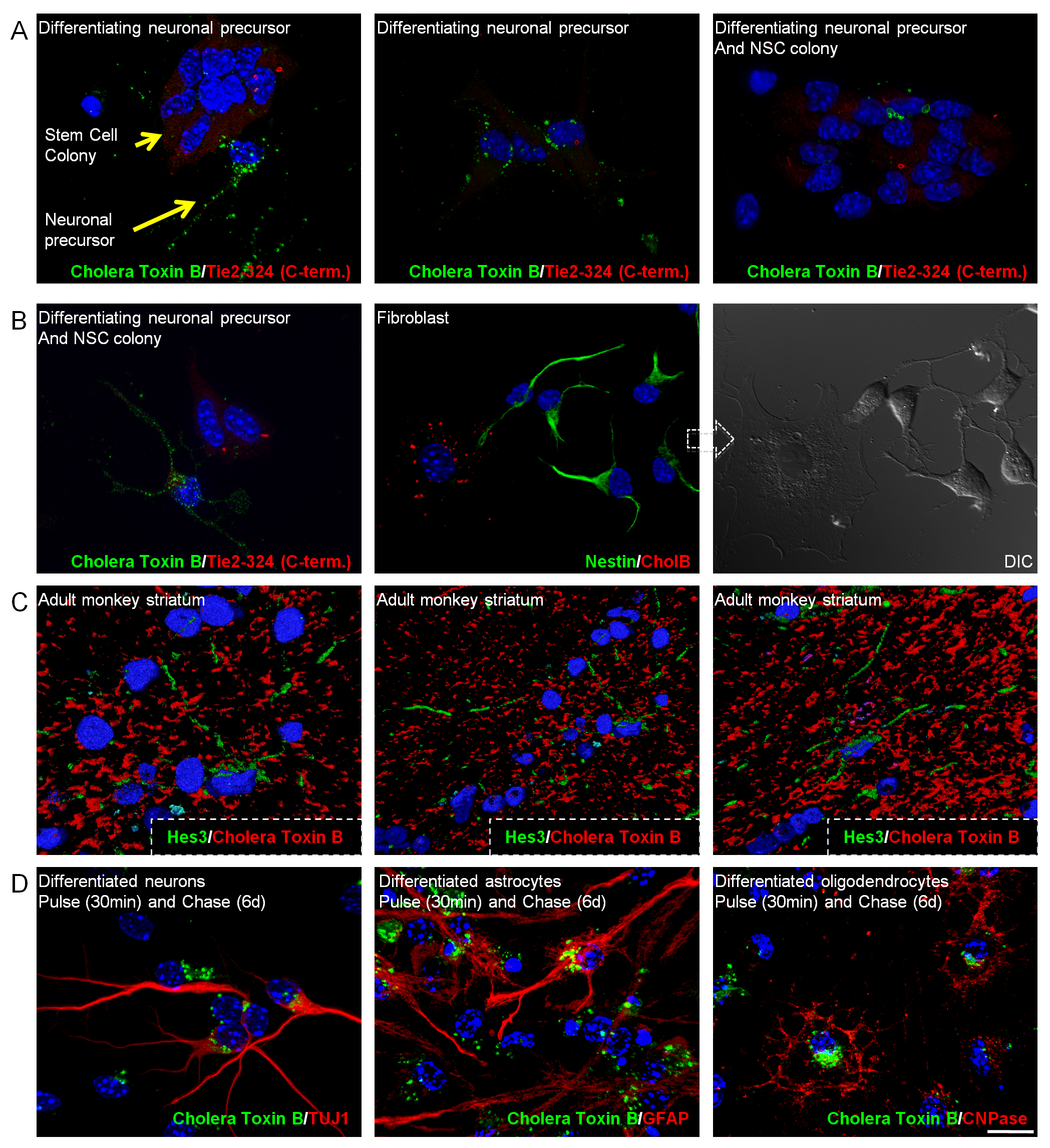

Supplement: Figure S1 — Cholera toxin labels differentiated cells in culture. (a,b) CholB labels differentiated DCX+ cells and fibroblast contaminants in culture; NSCs are identified by Tie2 immunoreactivity. (c) Adult monkey Hes3+ striatal neural precursors are not labeld by CholB. (d) A 30 min pulse of CholB in fetal NSC cultures 2 days after FGF2 withdrawal labels neurons, astrocytes and oligodendrocytes (chased for 6-days). [Size bars: 20 µm]. (3.43 MB TIF) [file pone.0010841.s001.tif]

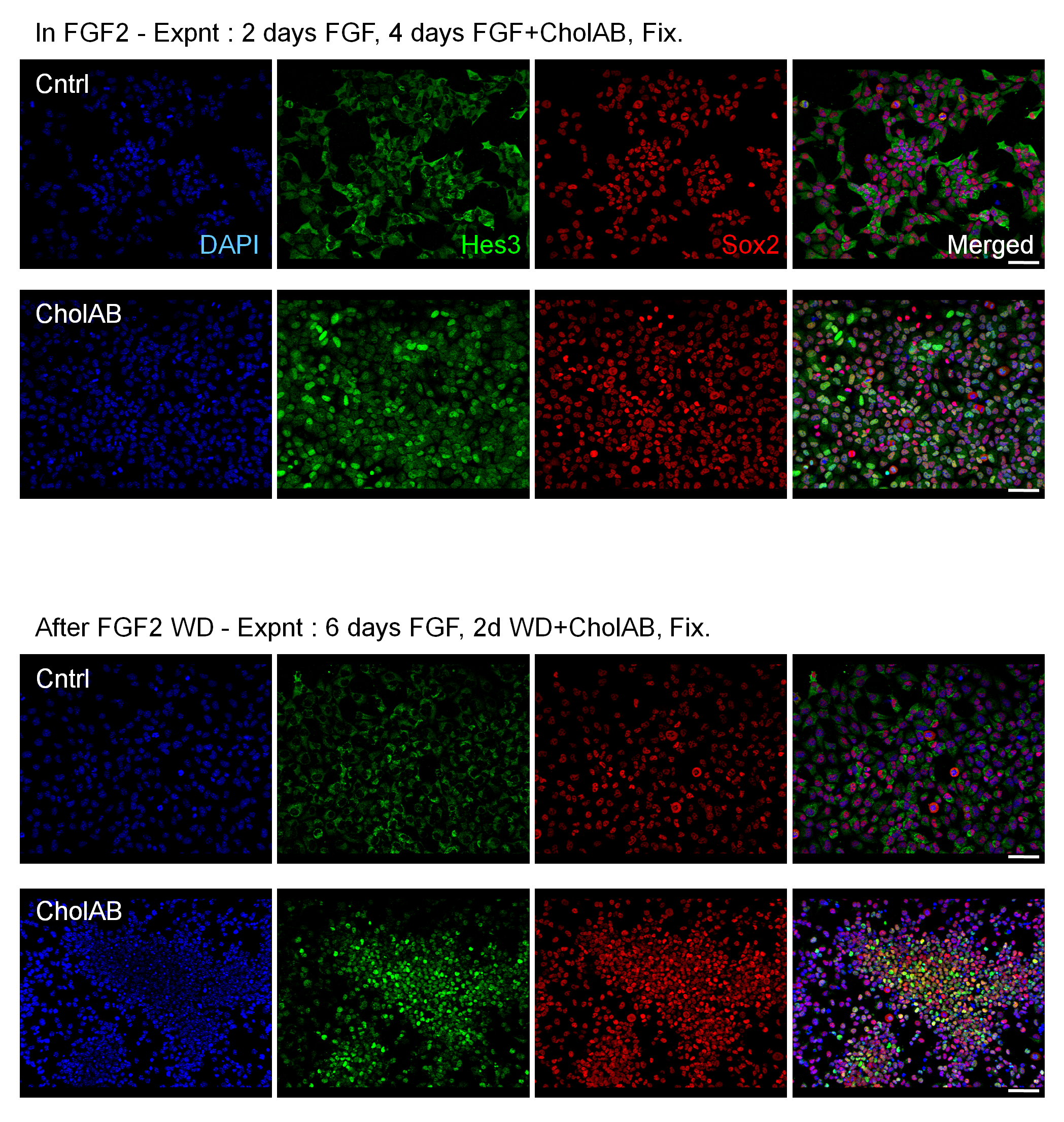

Supplement: Figure S2 — Cholera toxin promotes the nuclear localization of Hes3. CholAB (2-d treatment) promotes the nuclear localization of Hes3 in the presence and absence of FGF2 in fetal NSC cultures; split channels from confocal projections are shown [Size bars: 100 µm]. (3.79 MB TIF) [file pone.0010841.s002.tif]

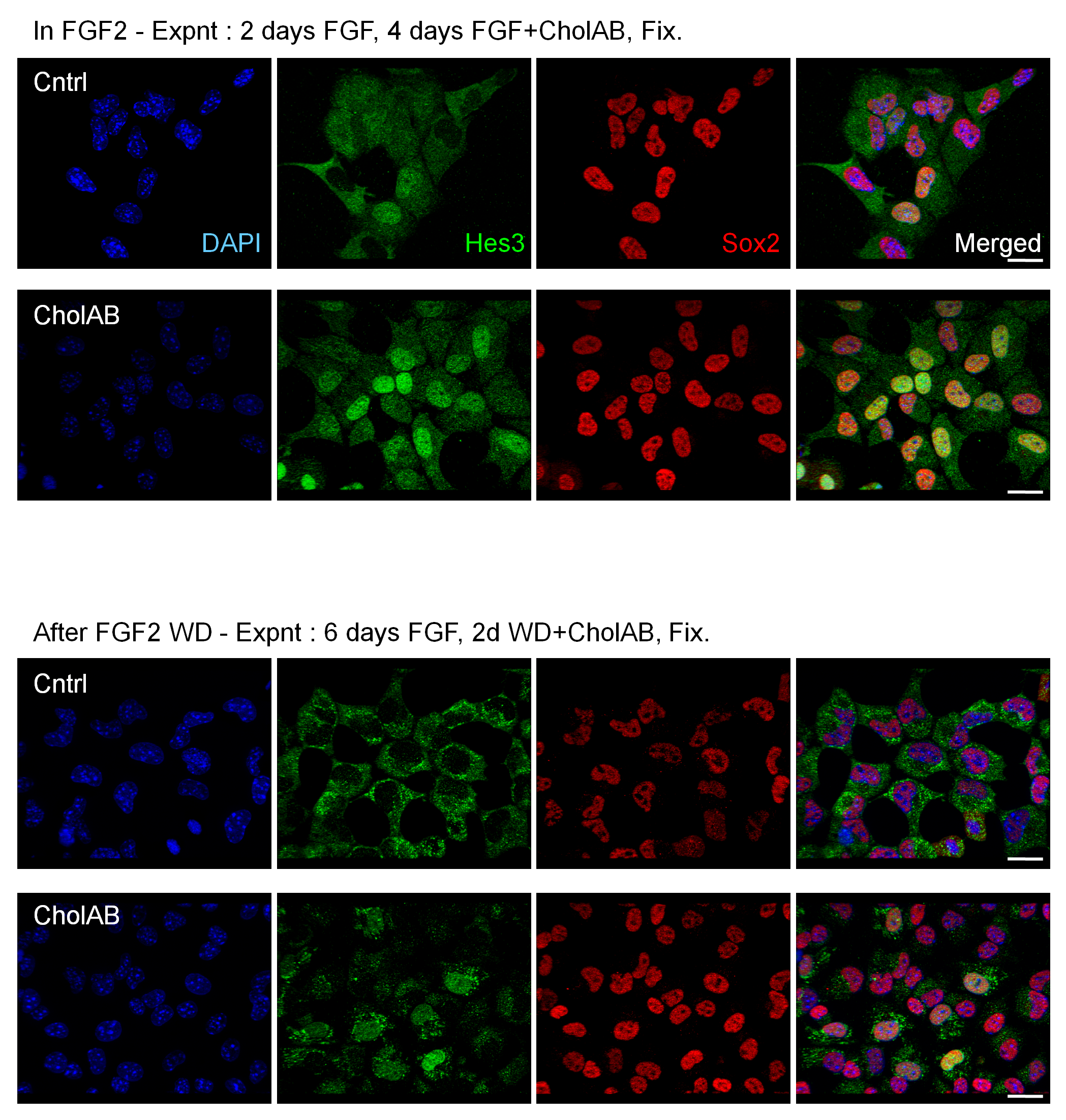

Supplement: Figure S3 — Cholera toxin promotes the nuclear localization of Hes3. High-power magnification of the images described in Suppl. Fig. S2 [Size bars: 20 µm]. (2.89 MB TIF) [file pone.0010841.s003.tif]

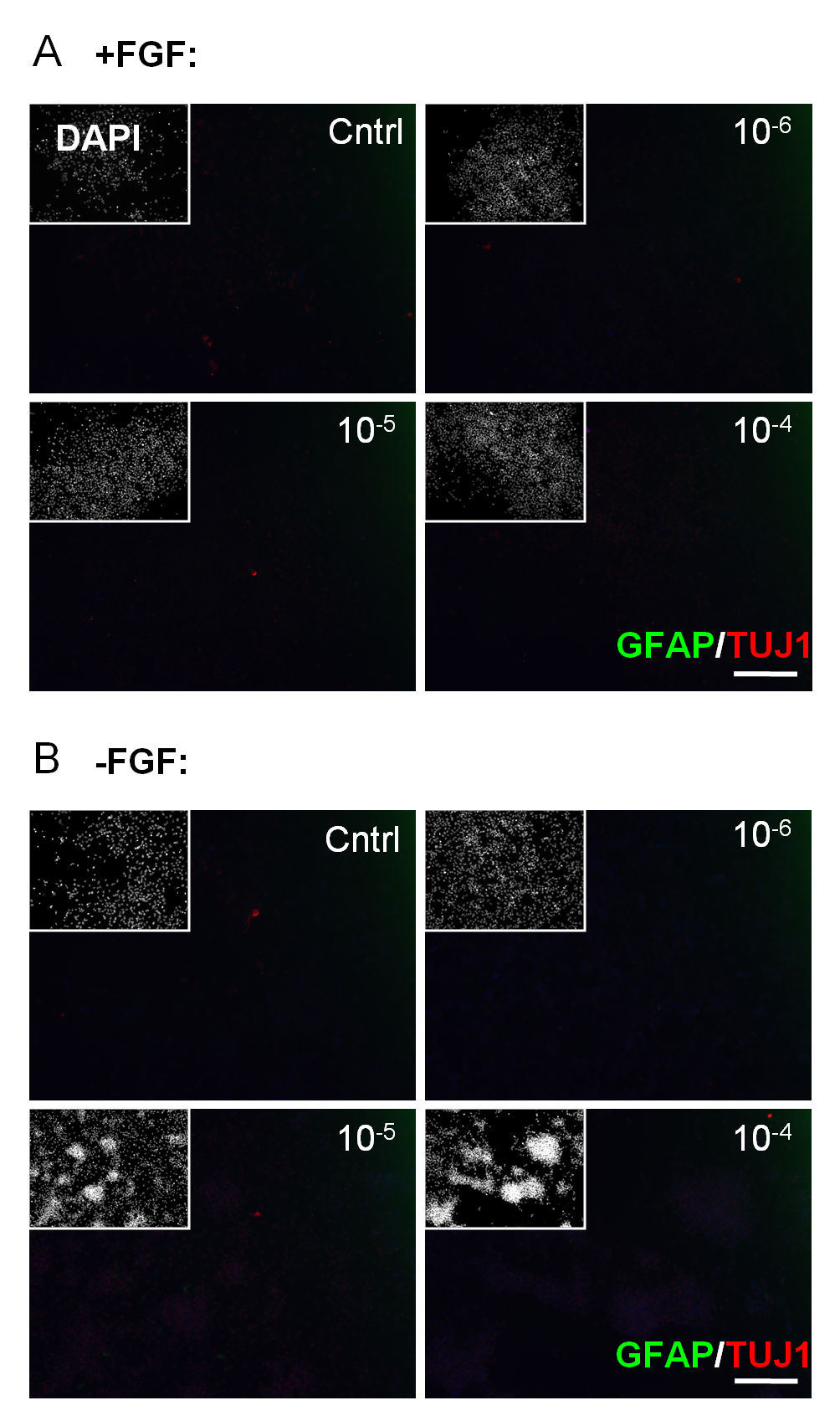

Supplement: Figure S4 — Cholera toxin inhibits the differentiation of fetal NSCs in culture. (a) Fetal NSC cultures expanded in FGF2 (4-days) and then switched to FGF2+/− CholAB - containing medium do not acquire immuno-reactivity for GFAP or TUJ1. (b) Fetal NSC cultures expanded in FGF2 (6-days) and then switched to +/− CholAB conditions, in the absence of FGF2 (for 2-days) do not acquire immuno-reactivity for GFAP or TUJ1. [Size bars: 200 µm]. (1.34 MB TIF) [file pone.0010841.s004.tif]
