# Supplementary material for: Cholera Toxin Regulates a Signaling Pathway Critical for the Expansion of Neural Stem Cell Cultures from the Fetal and Adult Rodent Brains
Source: PLoS One. 2010 May 26;5(5):e10841. doi: 10.1371/journal.pone.0010841 (PMC2877108; doi:10.1371/journal.pone.0010841)
Supplement: Table S1 — Significance (p) values. (0.04 MB DOC) [file pone.0010841.s005.doc]

**Supplementary Table 1. Significance (p) values.**

| **Figure** | **p Value** | **Description** |
| --- | --- | --- |
| 2c | ChAB (10^-5^) from Control: 0.01705452 | Adult Tie2 spike occurrence |
| 3a | In FGF2: ChAB (10^-5^) from Control: 1.1x10^-9^  After FGF WD: ChAB (10^-5^) from Control: 1.3x10^-8^ | % nuclear Hes3 localization |
| 4a | ChAB (10^-5^) from Control: 0.00091603  ChAB (10^-4^) from Control: 0.00064197  ChAB (10^-6^) from Control: 0.016477885  ChAB (10^-5^) from Control: 0.000414546  ChAB (10^-4^) from Control: 0.001477029 | Total cell# in FGF2  DCX+ cell# in FGF2 |
| 4b | ChAB (10^-6^) from Control: 0.004664756  ChAB (10^-5^) from Control: 0.000409248  ChAB (10^-4^) from Control: 0.025725397  ChAB (10^-6^) from Control: 0.027601212  ChAB (10^-5^) from Control: 0.01971878  ChAB (10^-4^) from Control: 0.103774835 | Total cell# after FGF2 Withdrawal  DCX+ cell# after FGF2 Withdrawal |
| 5b | ChAB (10^-5^) from Control: 0.0.00117645 | % EdU+ cells after FGF2 withdrawal |
| 6 | CT from Control: 0.000391933  ChAB from Control: 0.000631366  CT+ChAB from Control: 0.000622539  CT+ChAB from CT: 0.001653814  CT+ChAB from ChAB: 0.000828695 | Total cell# in FGF2 |
| 7d | ChAB from Control: 0.01512623 | Total cell# in FGF2 (Adult NSCs) |
